# The association between school violence and physical child-to-parent violence: is exposure to parental violence a moderator?

**DOI:** 10.1186/s12889-026-29657-3

**Published:** 2026-09-26

**Authors:** Robert Svensson, Lisa Andersson, Björn Johnson

**Affiliations:** 1 Department of Criminology, Malmö University, Malmö, 205 06 Sweden; 2 School of Social Work, Lund University, Lund, Sweden

**Keywords:** CPV, Physical CPV, School violence, Parental violence, Interaction

## Abstract

**Background:**

Child-to-parent violence (CPV) is a serious but under-researched form of family violence. There is a lack of previous studies examining whether school violence and exposure to parental violence interact in their association with CPV. We hypothesize that involvement in school violence – as victim or perpetrator – is more strongly associated with physical CPV among individuals who have been exposed to parental violence.

**Methods:**

Data are drawn from a large cross-sectional study conducted in 35 schools in southern Sweden. The sample comprises 5,052 adolescents (46.7% male; *M*age = 16.2, SD = 1.55). Linear probability models are used to calculate the associations between school violence, exposure to parental violence, and physical CPV.

**Results:**

The results show that involvement in school violence – either as a victim, perpetrator, or victim/perpetrator – is positively associated with physical CPV. The results support the hypothesis that the association between involvement in school violence and physical CPV is stronger among those individuals who have been exposed to parental violence.

**Conclusions:**

This study underscores the importance of further exploring how different contexts interact in their associations with CPV. These interactions suggest that a key question for future research is how to develop effective preventive strategies.

## Background

Over the past 15 years, there has been an increase in the number of empirical studies focusing on the violence committed by children and adolescents against their parents [1, 2]. Child-to-parent violence (CPV) has been defined as “repeated acts of physical, psychological (verbal or non-verbal), or economic violence perpetrated by children against their parents or parental figures” [3]. Definitions differ somewhat in scope, however: while some emphasize repetition as a defining criterion [3], others also include single acts if they are sufficiently severe [4]. CPV encompasses a variety of behaviors designed to control, coerce, or dominate the parent [5]. Although psychological violence, including verbal abuse, is the most common form of CPV [6], physical violence represents a more serious form of behavior, for which even an isolated incident can be considered abusive [4]. Research shows that the proportion of young people who report having used physical violence against a mother or father varies between 5 and 21% [6–8]. The present study focuses on physical CPV during adolescence – a developmental period during which such violence is considered a distinct problem behavior rather than age-typical aggression. It is based on a sample of students in compulsory and upper secondary school (aged 13–20). Specifically, we examine the association between physical CPV and involvement in violence in another key context of adolescent life: school.

Some of the more important predictors of CPV are related to individual differences and family relationships. A number of reviews emphasize that most empirical studies have focused on individual factors such as high impulsivity, aggressiveness, low frustration tolerance, and attitudes towards aggression [6, 9, 10], and/or environmental factors mainly related to the family, such as dysfunctional family relationships, low family cohesion, and low affection [6, 9–13]. Less attention has been paid, however, to the broader context in which adolescents spend their everyday lives. It has been observed that the most reliable indicator of aggressive behavior towards parents is the existence of similar behavior in other situations [6]. In line with this, it has been argued that adolescents who are aggressive towards their friends are also more likely to be aggressive towards their parents [6, 14]. It is therefore highly likely that adolescents who are involved in violence in one context – such as school, where they spend a significant amount of time during the week – tend to have more positive attitudes towards violent acts in general [15]. However, it has been stressed that there is limited evidence on how multiple and cross-setting exposure to violence (ETV) shapes aggressive outcomes [15].

How common, then, is it for young people who use physical violence against their parents also to engage in violence against peers in the school context? To our knowledge, only a few empirical studies have focused on the overlap between children’s physical violence against their parents and violence in schools [15, 16]. Two such studies have found an association between school-violence victimization and physical violence towards parents, indicating that being a victim of school violence is associated with a greater likelihood of exhibiting physical violence towards parents [15, 16]. There are also theoretical reasons to expect such an association. For example, victimization can be a source of strain, and aggression towards parents may may be a displaced response to frustration and anger that cannot safely be directed at the perpetrator. Furthermore, victimization can contribute to the normalization of violence in everyday interactions.

Further, some studies have found other school-related factors to be associated with CPV. For example, CPV has been found to be associated with school bullying and teacher abuse, while a positive classroom environment and strong school attachment act as protective factors against CPV [6, 17, 18]. One important question is whether these associations are moderated by other significant predictors.

### Exposure to parental violence as a moderator

One of the best-known predictors of CPV is experience of parent-to-child violence (often referred to as “bidirectionality”) or exposure to other types of family violence. A large number of studies have found that many young people who perpetrate CPV have also been victimized by their parents [14, 15, 19–23]. In the present study, exposure to parental violence refers to physical violence by a parent directed at the young person or their sibling. Despite the well-established link between parental violence and CPV, whether the relationship between school violence (as a victim or offender) and physical CPV varies with adolescents’ exposure to parental violence is not well understood.

Based on previous findings showing an association between school violence and CPV, it is reasonable to assume that there will be a positive association between experience of school violence (as a victim or perpetrator) and physical CPV. Since exposure to parental violence is such an important predictor of CPV, it is highly likely that the association between school violence and physical CPV will be dependent on the level of exposure to parental violence. This would lead to the association between school violence and physical CPV being stronger for individuals who have been exposed to parental violence. Such an overlap between different contexts, and a moderating effect of exposure to parental violence, would be in line with the social-ecological model [24], which argues that different social contexts (such as the family and the school) are important for the development of young people.

This argument is furthermore also in line with differential association theory [25] and social learning theory [26], which argue that delinquency (in this case violence) is learned in close relationships through social interaction. The responsible mechanisms include the transmission of delinquent [violent] definitions (norms, values, skills, and techniques), observation and imitation, and differential social reinforcement [26, 27]. Although peers are often highlighted as key agents in such learning processes, especially during adolescence, parents also play an important role in these processes, particularly during early childhood. Similar relationships have also been discussed in the light of the “cycle of violence” theory, which argues that violence breeds violence, i.e., that children who live in a violent home atmosphere are at increased risk of subsequently engaging in physical violence themselves [28].

To our knowledge, only one study has examined whether there is an interaction between being a perpetrator of school violence and exposure to parental violence in relation to the likelihood of engaging in CPV [15]. This study showed that the association between being a perpetrator of school violence and CPV was stronger for those who had been exposed to parental violence. No previous study has conducted a detailed examination of whether different types of exposure to school violence – as perpetrator, victim, or both perpetrator and victim – are differentially associated with physical CPV depending on the level of exposure to parental violence.

This study tests the following hypotheses:

- Hypothesis 1: Involvement in school violence (as a victim, perpetrator, or victim/perpetrator) is positively associated with physical child-to-parent violence.
- Hypothesis 2: There is an interaction between school violence (as a victim, perpetrator, or victim/perpetrator) and exposure to parental violence, such that the association between school violence and physical child-to-parent violence is stronger for individuals who have been exposed to parental violence.

## Method

### Participants

This study is cross-sectional and based on an online questionnaire among a stratified sample of school-aged adolescents and young adults, aged 13 to 20 (mean = 16.2 years, SD = 1.55), attending years 8 and 9 in compulsory school (normally ages 13–15) and years 1 to 3 in upper secondary school (normally ages 16–19). A small number of respondents (*n* = 90) were aged 20, in most cases due to delayed school entry. The vast majority of participants lived with their parents; the proportion living alone was 0% among the 13-year-olds, 2% among the 19-year-olds, and 17% among the 20-year-olds.

The questions used in this study are from a larger project on child-to-parent violence. The questions are based on well-established instruments. For detailed information about the questions see Supplemental appendix. The data were collected during the periods March-June and August-October 2022. In total, 305 classes from 35 schools in 12 municipalities in the south of Sweden participated. The classes comprised a total of 6,965 students, of whom 5,780 were present on the day of the survey. The relatively high rate of non-attendance, 17.0%, was due to pandemic guidelines that required students to stay at home if they were showing symptoms of respiratory infection. Of the students who were present, 5,352 completed the questionnaire, resulting in a total response rate of 92.6%. Total loss due to unreliable survey completion was 42 responses, leaving 5,310 responses that constitute the study’s dataset. For a detailed description of sampling processes, stratification, and data collection, see [4]. To examine whether there were any problems with non-random missing data, the Little MCAR test was performed. The test was significant and the Expectation-Maximization (EM) algorithm was used for imputation to handle missing data. The final sample used for the regression analyses consisted of 5,052 respondents (males 46.7%; born in Sweden 83.3%, born in another European country 4.3%, born in a country outside Europe 12.3%).

### Ethics approval and consent to participate

The study was reviewed by the Swedish Ethical Review Authority (reference number 2021-05901-01). The authority determined that since the study does not involve the handling of personal data in a manner that allows for linking the data to identifiable individuals, the study does not fall within the scope of the Swedish Ethical Review Act (SFS 2003:460). Consequently, the authority issued an advisory opinion, which we adhered to. Informed consent was obtained from all participants. For children under 15 years of age, informed consent was also obtained from their legal guardians, in accordance with Swedish research practice.

### Measures

#### Dependent variable

##### Physical child-to-parent violence

Is measured using a scale focused on physical violence against parents, which is based on three items from the ABC-I scale [6]. For a discussion of the validity of this measure, see [29]. For a validation of the Swedish version of this scale, see [4]. Participants were asked to indicate whether they had behaved in a particular way towards a parent during the past 12 months: (1) kept [a parent] from seeking help or medical care, (2) threatened [a parent] with an object, (3) acted physically aggressively towards [a parent] (e.g., pushed, grabbed, punched, kicked, burned, strangled, used weapon against, etc.). Responses were provided separately for each parent using the following alternatives: never / one time / a few times / every month / every week / daily. Cronbach’s alpha for the scale is 0.80. The analyses in this study use the following categorization: 0 = no involvement in physical violence / 1 = engaged in physical violence one or more times. A score of 1 thus indicates that the respondent had engaged in any of the specified acts against his/her mother/father.

#### Independent variables

##### Victim of school violence

Measures whether the respondents had: been seriously hit, kicked, or exposed to other violence at school in the last 12 months. Response alternatives: no, never / yes, sometimes / yes, every month / yes, every week / yes, daily. A total of 91.5% of the respondents reported that they had not been the victims of school violence, while 8.5% reported that they had been victimized on one or more occasions. The majority of those victimized (7% of the total sample) reported the response category ‘yes, sometimes’, while more frequent victimization (monthly or more often) was rare (1.5%). For this study we decided to use a 0/1 measure: 0 = no school-violence victimization / 1 = victim of school violence at least once in the last 12 months.

##### Perpetrator of school violence

Measures whether the respondents had: hit, kicked, or used violence against someone else at school in the last 12 months. Response alternatives: no, never / yes, sometimes / yes, every month / yes, every week / yes, daily. A total of 93.9% of the respondents reported that they had not perpetrated any school violence, with 6.1% reporting that they had been perpetrators of school violence at least once. The majority of these (5% of the total sample) reported the category ‘yes, sometimes’, while more frequent perpetration was rare (1.1%). For this study we decided to use a 0/1 measure: 0 = no involvement in school-violence perpetration / 1 = perpetrated school violence at least once.

##### Combination of victim and perpetrator of school violence

The two measures of being a victim and a perpetrator of school violence were combined into a measure that focuses on the overlap between the two. The two items are positively correlated, r = .57, indicating that victims of school violence are more likely than others to be involved in school violence as perpetrators. The combined measure shows that 89.8% of the respondents had not been involved in violence either as victims or perpetrators (category 1), 4.1% had only been victims of school violence (category 2), 1.8% had only been perpetrators (category 3), and 4.3% reported having been both victims and perpetrators (category 4). This measure is used as a series of dummy variables using category 1 as the reference group.

##### Exposure to parental violence

Is measured using a single item reflecting whether the respondents’ parents had been aggressive towards them or their siblings: Has either of your parents ever pushed, hit, or used other violence against you or a sibling? Response alternatives: no / yes, against me / yes, against sibling(s) / yes, against, both me and my sibling(s). For the analyses presented in the article, the variable has been dummy coded as: 0 = no / 1 = yes, against me; yes, against sibling(s); yes, against both me and my sibling(s).

##### Externalizing and internalizing problems

Were measured using the Swedish version of the Strengths and Difficulties Questionnaire (SDQ) [30, 31], a widely used self-report questionnaire for assessing child mental health problems. The questionnaire consists of five subscales: conduct problems, emotional symptoms, hyperactivity, peer problems, and prosocial behavior. Each subscale comprises four items with 3-point response scales (not true / somewhat true / very true). Hyperactivity and conduct problems were combined into the broader scale *externalizing problems* (Cronbach’s alpha = 0.78). Peer problems and emotional symptoms were combined into the broader scale *internalizing problems* (Cronbach’s alpha = 0.68). The measures of internalizing and externalizing problems are correlated at *r* = .33.

##### Positive and negative parenting styles

Were measured using the Swedish version of the Parents as Social Context Questionnaire (PASCQ) [32]. The PASCQ is an instrument developed to measure six parenting dimensions: warmth, rejection, structure, chaos, autonomy support, and coercion [33]. Each dimension is measured using four items with 4-point response scales (not at all true / not very true / somewhat true / very true). The three dimensions of warmth, structure, and autonomy support were combined into the broader scale *positive parenting style* (hereafter: PASCQ^Pos^) (Cronbach’s alpha = 0.91). The three dimensions of rejection, chaos, and coercion were combined into the broader scale *negative parenting style* (hereafter: PASCQ^Neg^) (Cronbach’s alpha = 0.91). Although these styles can be seen as opposites, the dimensions involved are dynamic, meaning that high scores on one dimension do not automatically imply low scores on the opposite dimension [32, 33]. They are correlated at *r* = − .51.

Sex is coded as 0 for females and 1 for males.

Descriptive statistics for the different measures are presented in Table 1.

**Table 1:** Descriptive statistics

	Mean/%	SD	Min	Max
Physical CPV	5.5%	-	0	1
School-violence victim	8.5%	-	0	1
School-violence perpetrator	6.1%	-	0	1
Categories of school violence				
No violence involvement	89.8%	-	0	1
Victim only	4.1%	-	0	1
Perpetrator only	1.8%	-	0	1
Victim & perpetrator	4.3%	-	0	1
Parental violence ^a^	12.4%	-	0	1
Externalizing problems	3.39	0.84	1	6
Internalizing problems	3.48	0.76	1	6
PASCQ^Neg^	1.78	0.64	1	4
PASCQ^Pos^	3.51	0.50	1	4
Sex (1 = male)	46.7%	-	0	1

^a^ Exposure to parental violence

### Analytical strategy

We estimated several *linear probability models* (LPM) with robust standard errors to examine our two research questions, i.e., to examine the association between school violence as a victim, perpetrator, and victim/perpetrator, and physical CPV, and to examine whether this association may be different for different individuals depending on their level of exposure to parental violence. The LPM estimates are comparable across groups [34] and are preferable to non-linear models when analyzing interactions [35]. The LPM estimates are interpreted as the percentage point change in the outcome probability given a unit increase in the independent variable [36].

The LPM regressions were estimated in two stages. First, we examined whether there is an association between school violence – both as victim and perpetrator – and physical CPV. Second, we examined whether there is an association between the different categories of school-violence experience – only victim, only perpetrator, both victim and perpetrator – and physical CPV. In both stages we examined whether the different types of school-violence experience interact with exposure to parental violence in the association with physical CPV. In most of the regression models we adjust for sex, externalizing problems, internalizing problems, and positive and negative parenting styles.

Finally, to illustrate the interactions, we plotted the average marginal effects using the *marginsplot* command in Stata. All statistical analyses have been conducted using Stata version 17.

## Results

To examine our research questions, i.e., whether school violence – as a victim or perpetrator – is associated with physical CPV, we present several regression models in Table 2. The results presented in Models 1 and 2 show that experience of being either a victim or a perpetrator of school violence is positively associated with physical CPV. According to the linear probability estimate, the association is slightly stronger for perpetrators of school violence than for victims.

**Table 2:** Association between physical CPV and being a victim or perpetrator of school violence

	Model 1		Model 2		Model 3		Model 4	
	Coef.	*p*	Coef.	*p*	Coef.	*p*	Coef.	*p*
Victim	0.080	< 0.001			0.039	0.013		
Perpetrator			0.105	< 0.001			0.056	0.006
Parental violence ^a^					0.095	< 0.001	0.100	< 0.001
Victim × Parental violence ^a^					0.150	0.005		
Perpetrator × Parental violence ^a^							0.184	0.003
Externalizing problems	0.020	< 0.001	0.018	< 0.001	0.020	< 0.001	0.018	< 0.001
Internalizing problems	− 0.004	0.338	− 0.002	0.720	− 0.004	0.335	− 0.002	0.696
PASCQ^Neg^	0.040	< 0.001	0.041	< 0.001	0.033	< 0.001	0.030	< 0.001
PASCQ^Pos^	− 0.048	< 0.001	− 0.051	< 0.001	− 0.028	0.005	− 0.030	0.002
Sex (1 = male)	− 0.013	0.039	− 0.015	0.022	− 0.008	0.245	− 0.010	0.147
R^2^	0.074		0.075		0.105		0.108	
N	5,052		5,048		5,022		5,018	

Linear probability model using robust standard errors. Child-to-parent violence measured as physical violence

^a^ Exposure to parental violence

Models 3 and 4 include exposure to parental violence and the interaction terms between exposure to parental violence and experience of being a victim or perpetrator of school violence. The results show that both interaction terms are positive and significantly associated with physical CPV. This indicates that the association between physical CPV and both school-violence victimization and having perpetrated school violence is significantly stronger among respondents who report that their parents had used violence against them or against their siblings.

In all models we adjust for sex, externalizing problems, internalizing problems, and positive and negative parenting. The measures of externalizing problems and both negative (PASCQ^Neg^) and positive (PASCQ^Pos^) parenting were all significantly associated with physical CPV in the expected direction. Sex is negatively and significantly associated with physical CPV in Models 1 and 2. 

**Fig. 1:**
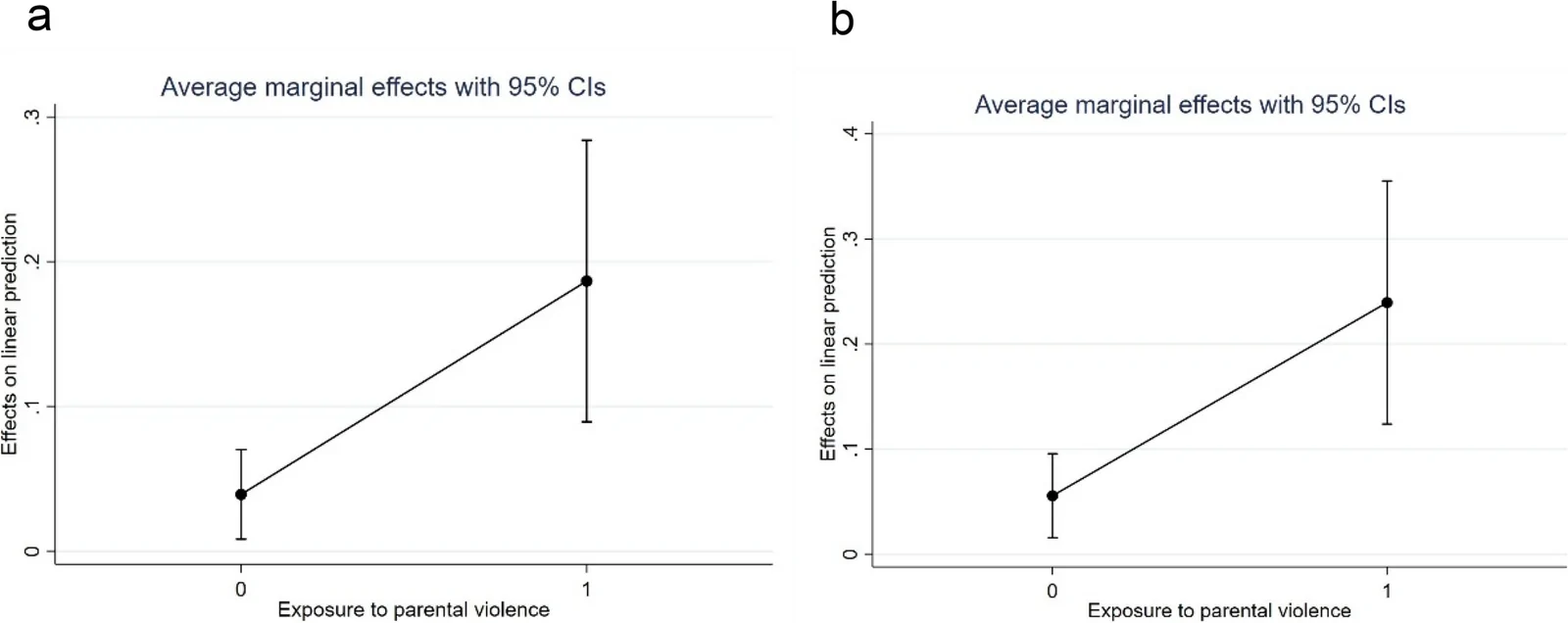
Association between physical CPV and (**a**) school-violence victimization, **b** school-violence perpetration, by exposure to parental violence (0 = no exposure; 1= exposure). Controls are included

To illustrate the interaction effects between violence in school and parental violence, we present the different estimates for the interactions in Fig. 1. The findings show that the association between physical CPV and being a victim or perpetrator of school violence is stronger for those who report parental violence (Fig. 1a and b). The association between being a victim of school violence and physical CPV is 0.039 (0.008, 0.070) for those with no exposure to parental violence, compared to 0.187 (0.089, 0.284) for those reporting experience of parental violence. The association between being a perpetrator of school violence and physical CPV is 0.056 (0.016, 0.096) for those with no experience of parental violence, compared to 0.239 (0.123, 0.355) for those who report experience of parental violence.

Finally, we estimated regression models to examine whether the three different categories of school-violence experience – only victim, only perpetrator, and both victim and perpetrator – were associated with physical CPV. Table 3 presents the results for these three categories, using those with no involvement in school violence as the reference category. The results show that membership of all three categories of school-violence experience was positively associated with physical CPV (Model 1).

**Table 3:** Association between physical CPV and different categories of school-violence experience

	Model 1		Model 2		Model 3		Model 4	
	Coef.	*p*	Coef.	*p*	Coef.	*p*	Coef.	*p*
Categories of school violence (ref: no violence)								
Victim only	0.081	< 0.001	0.054	0.017	0.047	0.032	0.041	0.054
Perpetrator only	0.116	0.003	0.095	0.011	0.094	0.013	0.093	0.018
Victim & perpetrator	0.155	< 0.001	0.111	< 0.001	0.102	< 0.001	0.042	0.071
Parental violence ^a^					0.116	< 0.001	0.094	< 0.001
Victim only × Parental violence ^a^							0.039	0.582
Perpetrator only × Parental violence ^a^							0.010	0.937
Victim & perpetrator × Parental violence ^a^							0.232	0.001
Externalizing problems			0.017	< 0.001	0.017	< 0.001	0.018	< 0.001
Internalizing problems			− 0.003	0.527	− 0.003	0.574	− 0.003	0.526
PASCQ^Neg^			0.040	< 0.001	0.029	< 0.001	0.030	< 0.001
PASCQ^Pos^			− 0.049	< 0.001	− 0.030	0.002	− 0.030	0.003
Sex (1 = male)			− 0.017	0.009	− 0.011	0.071	− 0.011	0.083
R^2^	0.028		0.077		0.101		0.110	
N	5,039		5,031		5,001		5,001	

Note: Linear probability model using robust standard errors. Child-to-parent violence measured as physical violence

^a^ Exposure to parental violence

Of the three school-violence categories, being both a victim and perpetrator of school violence was the most strongly associated with physical CPV. The results are stable when adjusting for sex, externalizing problems, internalizing problems, and positive (PASCQPos) and negative (PASCQNeg) parenting in Model 2. 

In Model 3, exposure to parental violence is also added to the model. As expected, parental violence is positively associated with physical CPV, indicating that young people who are exposed to parental violence are more likely to abuse their parents. Of the three school-violence categories, physical CPV is again most strongly associated with being both a victim and a perpetrator of school violence. 

Finally, in Model 4, we add the interaction terms between exposure to parental violence and the three categories of school violence. The results show that the only significant interaction is that between exposure to parental violence and being both a victim and perpetrator of school violence. This indicates that being both a victim and perpetrator of school violence is more strongly associated with physical CPV among those who have experience of parental violence.

**Fig. 2:**
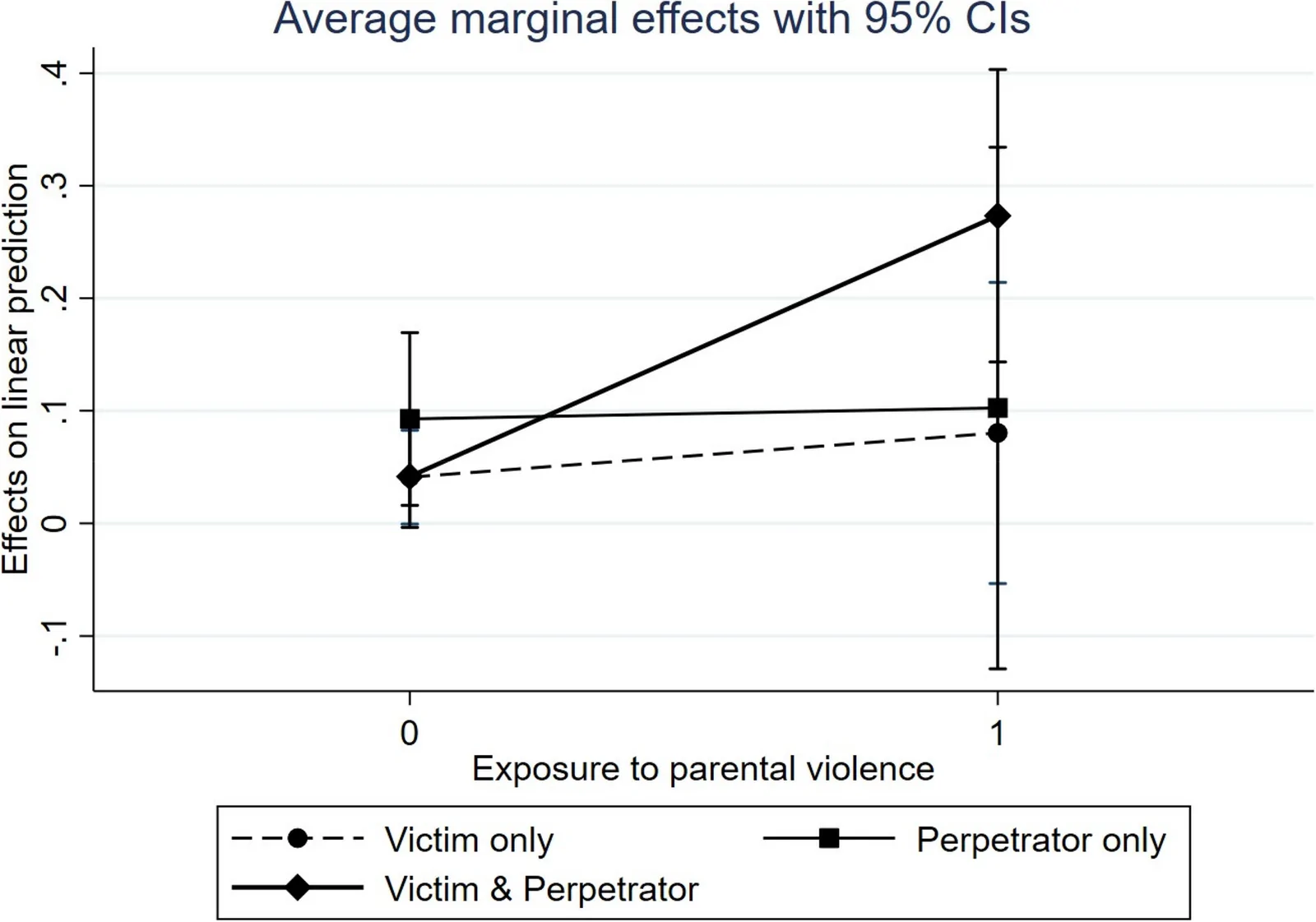
Association between three categories of school-violence experience and physical CPV by exposure to parental violence (0=no exposure; 1= exposure). Baseline: no involvement in school violence. Controls are included

To illustrate the different interaction effects between violence in school and parental violence we present the different estimates for the interactions in Fig. 2. The results show that the association between physical CPV and being both a victim and perpetrator of school violence is stronger for those reporting experience of parental violence than for those with no experience of parental violence. The association between physical CPV and having been both a victim and perpetrator of school violence is 0.042 (-0.004, 0.087) for those with no experience of parental violence, compared to 0.273 (0.143, 0.403) for those who report having experienced parental violence.

## Discussion and conclusion

This study has examined the association between school violence as a victim, perpetrator, and victim/perpetrator and physical child-to-parent violence in a large-scale cross-sectional study in the south of Sweden, and has also studied whether this association may be different for different individuals depending on their level of exposure to parental violence. Although there has been considerable discussion of the importance of examining the overlap between violence in different contexts, not many studies have examined the overlap between school violence and child-to-parent violence [6].

First, we found that being either a victim or perpetrator of school violence was positively associated with physical CPV. This finding is in line with previous research reporting that young people who are victims of school violence also abuse their parents at home to a greater extent than others [16]. For the victim role, this may reflect strain processes, whereby aggression towards parents functions as a displaced response to victimization that cannot safely be directed at the perpetrator. For the perpetrator role, our findings support the central theoretical argument that being violent in one context increases the probability of also being violent in other contexts [6]. In an extended analysis, we examined the association between physical CPV and different categories of school-violence experience (as a victim, perpetrator, or both a victim and a perpetrator). We found that all three categories of school-violence experience were significantly associated with physical CPV. It is interesting to note that the associations are slightly stronger for those who are both victims and perpetrators.

Second, we found that the relationships between our measures of school violence and physical CPV were moderated by levels of exposure to parental violence. This is an interesting finding, and indicates that exposure to parental violence seems to be a key factor in the process of becoming involved in CPV. Previous studies have also found evidence of this interplay for perpetrators of school violence [15].

Finally, our findings show that the association between school violence and physical CPV is most clearly moderated by exposure to parental violence among young people who report being both perpetrators and victims. It should be noted that the perpetrator-only category is small (1.8% of the sample), and the absence of a significant interaction for this group may partly reflect limited statistical power. For the combined group, however, the literature on school bullying offers some guidance. A number of empirical studies and theoretical discussions suggest that bully/victims display a more complex and severe problem profile than individuals who are only bullies or only victims. This includes more aggressive and externalizing behavior, more depressive symptoms, and more behavioral problems [37, 38]. If similar differences apply in the context of school violence, adolescents who are both victims and perpetrators may respond particularly strongly to conflict situations at home, especially if violent parent–child interaction is already part of the family environment. This may explain why the interaction with exposure to parental violence was found only for this group. In conclusion, these findings suggest that it is the combination of school violence and exposure to parental violence, rather than these factors in isolation, that most increases the likelihood of physical CPV. Further research is needed to test these interpretations.

Theoretically, our findings indicate that it is important to examine the overlap between different contexts in which violence occurs, and school and the family are assumed to be two important contexts in relation to young people’s development. Finally, the overlap found between the violence that occurs in different contexts provides some support for the ecological perspective [24], which argues that individuals are exposed to different contexts, which all have an impact on young people. Of the predictors included in this study, however, we found that the main predictor of physical child-to-parent violence was exposure to parental violence – which is a well-established finding in the field of physical child-to-parent violence. Our findings are also in line with the theoretical statements of the learning perspective, i.e., that spending time in a context where violence is common increases the likelihood of engaging in violence oneself.

Although the cross-sectional design precludes conclusions about temporal order, the theoretical perspectives outlined above suggest a plausible chain of events. Exposure to parental violence, which typically begins in early childhood, may contribute to the learning and normalization of violence as a strategy for conflict management. This increases the risk of involvement in violence in other contexts, such as at school. Among adolescents with a history of violent parent–child interaction, such involvement is, in turn, more likely to be accompanied by physical violence against parents. Our finding that the association between school violence and physical CPV is stronger among those exposed to parental violence is consistent with this interpretation.

A key limitation of the study is that the data have been collected using a cross-sectional design, and we are thus unable to draw conclusions about causality. Therefore, it is not possible to determine with certainty what influences what. For instance, we cannot establish whether participation in school violence leads to physical CPV, or whether it is physical CPV that leads to school violence. Alternatively, the relationship may work in both directions, i.e. it may be ‘bidirectional’. It is therefore important for future research to replicate these findings using longitudinal data, which would make it possible to examine the temporal ordering of parental violence, school violence, and physical CPV.

This being said, we would argue that future research should examine the overlap between different contexts not only in relation to physical CPV, but also in relation to other forms of child-to-parent violence. We would also argue that more research is needed on the interaction between exposure to parental violence and school violence in relation to physical CPV. One key question to be addressed is how these findings can help us develop preventive strategies against this kind of child-to-parent violence – and whether the existence of interactions between contexts might complicate the development of such strategies. One challenge would be to decide where the prevention strategies should be focused. Previous research has shown that some prevention strategies focus on the individual’s propensity for violence, while others focus on the situation, such as the school environment. In our study, the association between school violence and physical CPV differed depending on individuals’ exposure to parental violence. Based on this, we argue that it might be important to develop preventive strategies for individuals to help them avoid committing violence, focusing on early prevention (i.e. family- and school-related strategies) [39, 40]. Moreover, the overlap between contexts suggests that the detection of violence in one domain – for example, school violence identified by school staff – may serve as an indicator of risk in the other, providing opportunities for earlier identification of families in need of support.

## Data Availability

The datasets used in the current study are not publicly available but are available from the corresponding author on reasonable request.
